# Quatramer™ encapsulation of dual‐targeted PI3‐Kδ/HDAC6 inhibitor, HSB‐510, suppresses growth of breast cancer

**DOI:** 10.1002/btm2.10541

**Published:** 2023-05-12

**Authors:** Sachchidanand Tiwari, Suiyang Liu, Mohd Anees, Neha Mehrotra, Ashish Thakur, Gregory J. Tawa, Gurmit Grewal, Richard Stone, Surender Kharbanda, Harpal Singh

**Affiliations:** ^1^ Centre for Biomedical Engineering Indian Institute of Technology Delhi New Delhi India; ^2^ Dana Farber Cancer Institute, Harvard Medical School Boston Massachusetts USA; ^3^ National Center for Advancing Translational Sciences National Institutes of Health Rockville Maryland USA; ^4^ Department of Biomedical Engineering All India Institute of Medical Sciences Delhi New Delhi India

**Keywords:** breast cancer, HDAC6, Nanoformulation, PI3‐Kδ, PLA

## Abstract

Multiple studies have shown that the progression of breast cancer depends on multiple signaling pathways, suggesting that therapies with multitargeted anticancer agents will offer improved therapeutic benefits through synergistic effects in inhibiting cancer growth. Dual‐targeted inhibitors of phosphoinositide 3‐kinase (PI3‐K) and histone deacetylase (HDAC) have emerged as promising cancer therapy candidates. However, poor aqueous solubility and bioavailability limited their efficacy in cancer. The present study investigates the encapsulation of a PI3‐Kδ/HDAC6 dual inhibitor into hybrid block copolymers (polylactic acid‐methoxy polyethylene glycol; polylactic acid‐polyethylene glycol‐polypropylene glycol‐polyethylene glycol‐polylactic acid) (HSB‐510) as a delivery system to target PI3‐Kδ and HDAC6 pathways in breast cancer cells. The prepared HSB‐510 showed an average diameter of 96 ± 3 nm, a zeta potential of −17 ± 2 mV, and PDI of ˂0.1 with a slow and sustained release profile of PI3‐Kδ/HDAC6 inhibitors in a nonphysiological buffer. In vitro studies with HSB‐510 have demonstrated substantial growth inhibition of breast cancer cell lines, MDA‐MB‐468, SUM‐149, MCF‐7, and Ehrlich ascites carcinoma (EAC) as well as downregulation of phospho‐AKT, phospho‐ERK, and c‐Myc levels. Importantly, bi‐weekly treatment of Balb/c wild‐type mice harboring EAC cells with HSB‐510 at a dose of 25 mg/kg resulted in significant tumor growth inhibition. The treatment with HSB‐510 was without any significant effect on the body weights of the mice. These results demonstrate that a novel Quatramer encapsulation of a PI3‐Kδ/HDAC6 dual inhibitor (HSB‐510) represents an approach for the successful targeting of breast cancer and potentially other cancer types.

## 1 INTRODUCTION

Breast cancer is the leading cause of cancer‐related deaths in women.^1^ However, early diagnosis and conventional therapies result in a significant decrease in mortality. Cancer initiation and progression depend on multiple receptors or signaling cascades, suggesting that the therapies with multitargeted anticancer agents may benefit cancer patients. For example, cisplatin combined with doxorubicin or paclitaxel is given to treat patients with inherited BRCA (BReast CAncer) gene mutations.^2^, ^3^ The combination of bromodomain and extra‐terminal motif (BET) inhibitor with PI3‐K inhibitor showed promising anticancer effects in metastatic breast cancer.^4^ Phosphoinositide 3‐kinases (PI3‐K) are a family of lipid kinase enzymes that transduce signals from cell surface receptors to downstream effectors of various cellular processes, including survival, proliferation, differentiation, metabolism, and angiogenesis.^5^ Studies have shown that inhibitors of PI3‐K block downstream AKT/mTOR pathway, which regulate cell growth.^6^, ^7^ Few examples of PI3‐K inhibitors for cancer therapy include Idelalisib, Copanlisib, Duvelisib, Umbralisib, and Alpelisib.^6^, ^8^

Histone deacetylase (HDAC) belongs to the family of epigenetic enzymes that regulate gene expression by removing acetyl groups from e‐amino lysin residue in the amino‐terminal tail of histones, making the surrounding DNA less accessible to transcription factors.^9^ HDAC regulates the expression of various cancer‐related proteins, including p53 and p21, through maintaining the transcription.^10^ HDACs are overexpressed in multiple cancers, including breast cancer, pancreatic ductal carcinoma, and prostate cancer.^11^ Several studies have demonstrated that inhibition of HDACs showed promising outcomes in cancer therapy.^10^, ^11^, ^12^ Examples of HDAC inhibitors for cancer therapy include Vorinostat (SAHA), Balinostat, Panobinostat, Romidepsin, and Chidamide.^13^, ^14^

Cancer treatment with a single PI3‐K or HDAC inhibitor is limited by insufficient efficacy and resistance. PI3K and ERK signaling pathways have been implicated in the protection of cells from oxidative stress generated from HDAC inhibitor treatment,^15^, ^16^ which resulted in cell survival. While the simultaneous inhibition of both PI3‐K and HDAC synergistically inhibits tumor growth.^17^, ^18^, ^19^, ^20^, ^21^ For example, BEZ‐235 (PI3‐K/mTOR) inhibitor combined with Trichostatin A (HDAC inhibitor) showed synergistic antitumor activity in breast cancer treatment.^22^ BEZ‐235 potentiates the activity of HDAC inhibitor (Panobinostat) in treating non‐Hodgkin lymphoma (NHL) through multifactorial synergism involving AKT inactivation, Bim up‐regulation, myeloid cell leukemia‐1 (Mcl‐1) down regulation and enhanced DNA damage.^23^ There are two different strategies often used to target multiple signaling cascades involved in cancer, treatment with the combination of two or more single‐targeted drugs or administration of the multitargeted single anticancer agent. Significant efforts have been made to treat various cancer types by combining two or more anticancer drug molecules to target multiple signaling pathways. The dose‐limiting toxicities and drug–drug interaction limit cancer treatment with two or more anticancer agents.^24^ Therefore, recent research has emerged to develop multitargeted drug molecules that can target two or more pathways to treat cancer. The multitargeted drug molecules can easily overcome the limitation of dose‐limiting toxicities and drug–drug interaction associated with single‐targeted drug and their combination therapies.^25^, ^26^ In this context, several PI3‐K/HDAC dual inhibitors have been developed for various cancer treatments.^27^, ^28^, ^29^, ^30^ Most of them showed in vitro and in vivo antitumor activities, but their efficacies are limited due to lower bioavailability after oral administration.^31^, ^32^, ^33^ Moreover, the poor water solubility of dual inhibitor anticancer agents limited their intravenous (i.v.) administration, resulting in faster excretion and lower tumor accumulation.^34^, ^35^, ^36^

Nanoparticle (NP) delivery systems based on biodegradable polymers offer several advantages over free drug molecules. It allows improved pharmacokinetics (PK) of the encapsulated drug molecule with longer blood circulation time, higher accumulation in the tumor with leaky blood vasculature, and poor lymphatic drainage by the enhanced permeability and retention (EPR) effect.^37^ Poor aqueous soluble and toxic drug molecules can be encapsulated in the polymeric system to improve bioavailability and efficacy.^38^ In addition, polymeric NPs can be tailored to block multidrug resistance (MDR)/P‐glycoprotein (Pgp) efflux pumps.^39^ For example, tri‐block copolymers of polyethylene glycol (PEG)‐polypropylene glycol (PPG)–polyethylene glycol (PEG), also known as poloxamers, are widely used in drug delivery systems due to their inhibitory activity on efflux pumps.^40^ Poloxamers or pluronics, such as L61, P105, P188, F127, and P85 interact with the cell membrane where drug efflux transporters are localized and change its properties, which are critical for the proper functioning of ATP‐binding cassette (ABC) transporters (Pgp, MRP, and BCRP).^41^, ^42^ Multiple micellar nanoformulations of various anticancer drugs have been evaluated in preclinical and clinical models, but their burst release and toxicity profiles led them to fail in clinical trials.^41^, ^43^ However, poloxamer (P105 and F127) modified biodegradable polymer‐based drug delivery systems have shown improved therapeutic efficacy as compared to micellar nanoformulations in various cancer types.^44^, ^45^

In the present study, poloxamer L‐61 modified polylactic acid block copolymers were synthesized to prepare isoform‐specific single agent PI3‐Kδ/HDAC6 dual inhibitor (Figure S1) encapsulated nanoformulation (HSB‐510) to treat breast cancer. The in‐vitro anticancer activity of HSB‐510 was evaluated in breast cancer cell lines. As a proof‐of‐concept, we have also assessed the in‐vivo/ex‐vivo biodistribution of indocyanine green (ICG)‐encapsulated NPs on EAC tumor‐bearing mice. Ehrlich ascites carcinoma (EAC) has been chosen as syngeneic breast cancer tumor model^46^, ^47^, ^48^, ^49^ to evaluate anticancer therapeutic efficacy of HSB‐510.

## 2 RESULTS

### 2.1 PLA block copolymer synthesis and characterization

**FIGURE 1:**
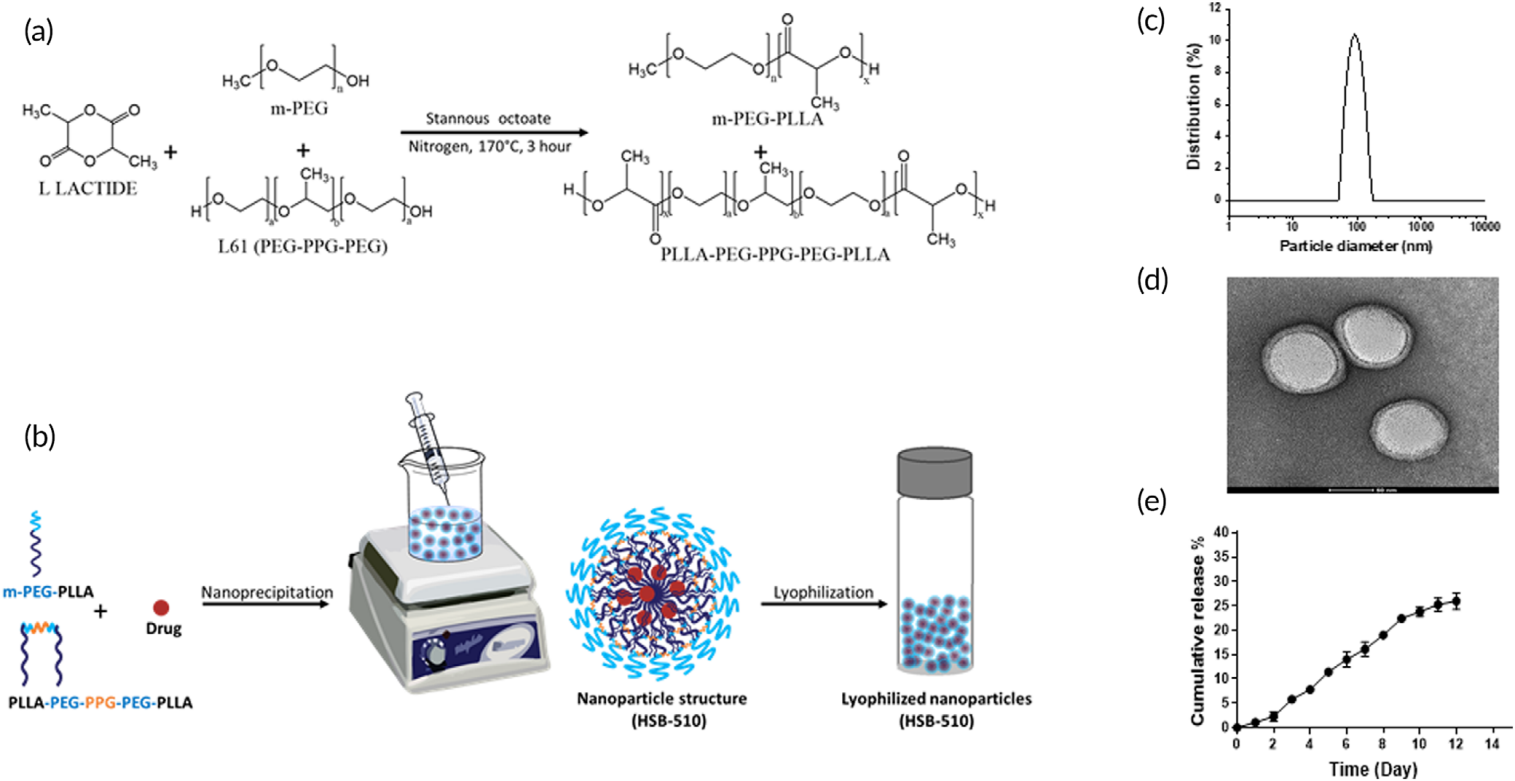
(a) Ring‐opening polymerization of l‐lactide using methoxy‐polyethylene glycol (mPEG) and L‐61 as initiator and stannous octoate as a catalyst at 170°C for 3 h under nitrogen purging. (b) Schematic diagram representing preparation of PI3‐Kδ/HDAC6 encapsulated block copolymeric nanoparticles (HSB‐510). (c) Hydrodynamic radius of HSB‐510 as determined by DLS. (d) High‐resolution transmission electron microscopy (HR‐TEM) image of HSB‐510 nanoformulation. (e) In vitro drug release study of HSB‐510 at physiological conditions (0.1 M PBS [pH 7.4], 37°C, 150 rpm).

Poloxamers are modulators of MDR and Pgp efflux pumps resulting in sensitization of drug‐resistant tumors.^50^, ^51^ MDR reversal activity of poloxamers depends on the content of hydrophobic and hydrophilic block length as it defines the interaction with cell and mitochondrial membrane.^41^ Multiple studies have demonstrated recently that poloxamer L‐61 based drug delivery systems could increase the cytotoxicity of drug molecules against resistant cancer cells.^42^, ^51^ However, their lower stability and faster drug release profile make them unsuitable for cancer treatment. In the present study, we developed a new drug delivery system based on biodegradable PLA block copolymers comprising L‐61 and mPEG. Four PLA‐based hybrid block copolymers comprising penta‐block and di‐block were synthesized via ring‐opening polymerization of l‐lactide in the presence of varying mole ratios of L‐61 and mPEG (Figure 1a). Synthesized block copolymers designated as PLA/L‐61_100_, PLA/L‐61_75_, PLA/L‐61_50_, and PLA/L‐61_25_ according to the content of penta‐block copolymer ratio in comparison with di‐block copolymer. These block copolymers were characterized for their molecular weight and polydispersity index (PDI) by gel permeation chromatography (GPC) (Table 1, Figure S2). As determined by the GPC, the molecular weight (Mw) of all block polymers is between 24 and 26 KDa with a narrow polydispersity index, that is, 1.2–1.4.

**TABLE 1:** Characterization of synthesized block copolymers.

Sample	Monomer (mmol)	Initiator (mmol)		GPC of block copolymers			Nanoparticle size (nm)	Zeta potential (mV)	Stability
	l‐Lactide	L‐61	mPEG	Mw	Mn	Mw/Mn			
PLA/L61_100_	69.44	0.462	—	25,456	18,115	1.405	144 ± 8	−31	Settled
PLA/L61_75_	69.44	0.347	0.115	26,383	22,099	1.194	86 ± 6	−12	Settled
PLA/L61_50_	69.44	0.231	0.231	26,221	22,626	1.159	72 ± 3	−10.2	Settled
PLA/L61_25_	69.44	0.115	0.347	24,008	19,952	1.2	62 ± 4	−9.3	Stable

*Note*: GPC of block copolymers were done at room temperature using Viscotek GPCmax system with tetrahydrofuran as mobile phase. Nanoparticles (NP) size and zeta potential were measured using DLS instrument (Anton Paar Lightsizer 500).

^1^H NMR of sample PLA/L‐61_100_ penta block copolymer showed doublet peak at ~1.13 ppm from protons of CH_3_ units of PPG blocks in L‐61 and other peaks at ~3.4 and ~ 3.5 ppm are due to the CH and CH_2_ units of PPG in L‐61 (Figure S3A). The sharp singlet at ~3.63 ppm was from protons of CH_2_CH_2_ units of PEG blocks in L‐61. Doublet peak at ~1.58 ppm represented CH_3_ proton in PLA and quartet peak at ~5.19 ppm was from CH proton of PLA available in penta‐block copolymer. ^1^H NMR of other samples, including PLA/L‐61_75_, PLA/L‐61_50_, and PLA/L‐61_25_ hybrid block copolymers showed extra peaks at ~3.37 ppm due to CH_3_ protons of methoxy polyethylene glycol present in di‐block copolymer (mPEG‐PLA) (Figure S3B–D). Moreover, after synthesis of di‐block copolymer with penta‐block copolymer, the intensity of singlet ~3.6 ppm increased due to protons of CH_2_CH_2_ in mPEG block.

### 2.2 Preparation and characterization of NPs

All four block copolymers were evaluated for the preparation of NPs through the nanoprecipitation method^52^, ^53^ (Figure 1b). The physicochemical properties of these NPs, such as hydrodynamic diameter, zeta potential, and stability, are shown in Table 1. PLA/L‐61_100_ block copolymer was synthesized only with L‐61 initiator and hence has only one type of block copolymer, that is, PLA–PEG–PPG–PEG–PLA. The PEG content or chain length in PLA/L‐61_100_ is small which makes the block copolymer hydrophobic. Therefore, the NPs prepared using PLA/L‐61_100_ block copolymer have shown the largest size (144 ± 8 nm) with least stability in aqueous phase. The hydrophilicity defines the polymeric NPs stability and in vivo circulation time.^54^ Three more block copolymers were synthesized with increasing hydrophilic (mPEG) content through one‐pot synthesis by dividing initiator mole ratio into two initiators (L‐61 and mPEG). The NPs prepared with hybrid block copolymer PLA/L‐61_75_ showed lower size 86 ± 6 nm due to the presence of hydrophilic mPEG‐PLA di‐block copolymer (25%). As we increased the content of mPEG‐PLA di‐block in hybrid block copolymer by increasing the mPEG mole ratio, the NPs size further decreased. For PLA/L‐61_50_ and PLA/L‐61_25_ block copolymer the NPs size was about 72 and 63 nm, respectively, which is much lower in comparison to NPs prepared with single penta‐block copolymer. As the block copolymer hydrophilicity increases, the NPs showed more stability in the aqueous phase (Table 1). Based on NPs stability evaluated based on their change in hydrodynamic size and settling in aqueous medium, we selected PLA/L‐61_25_ block copolymer synthesized with 25% L‐61 and 75% mPEG.

### 2.3 Preparation and characterization of HSB‐510

The PI3‐Kδ/HDAC6 dual inhibitor encapsulated NPs (HSB‐510) were prepared and characterized for their size and morphology by DLS and high‐resolution transmission electron microscopy (HR‐TEM). The measured DLS size of HSB‐510 was 96 ± 3 with PDI of 0.1–0.2 (Figure 1c), which is higher than the blank NPs prepared with PLA/L‐61_25_ block copolymer. This is due to the encapsulation of hydrophobic dual PI3‐Kδ/HDAC6 inhibitor inside the core of NPs. The HR‐TEM image of HSB‐510 (Figure 1d) showed a size range of 75–90 nm with spherical morphology and bilayer structure, that is, the inner part of the hydrophobic core and outer layer of the hydrophilic part interfacing with the aqueous medium. NPs of SAHA, IDL were also prepared and characterized the same way with the DLS. The size of SAHA‐NPs and IDL‐NPs were 71 ± 4 and 83 ± 7 nm, and the PDIs were 0.18 and 0.23, respectively. Rho‐B dye‐encapsulated NPs and ICG dye‐encapsulated NPs were prepared for in vitro internalization and in vivo biodistribution studies. Maximum drug encapsulation efficiency obtained for PI3‐Kδ/HDAC6 inhibitor was about 96% due to its hydrophobicity. Encapsulation efficiency for SAHA and IDL was about 54% and 78%, respectively. SAHA is comparably less hydrophobic among all three anticancer agents, so its lesser amount was entrapped into the hydrophobic core of the NPs. HSB‐510, SAHA‐NPs, and IDL‐NPs showed LC% of 8.7%, 5.1%, and 7.2%, respectively.

### 2.4 In vitro release studies

HSB‐510 showed a cumulative release of about 30% in 12 days (Figure 1e). In contrast, the release of SAHA and IDL encapsulated NPs showed cumulative release of 50%–65% in 12 days (Figure S4A,B). SAHA and IDL encapsulated NPs showed burst release in the initial days, followed by a sustained release of 3%–6% for up to 12 days. Burst release of the drug from the NPs could be due to the adsorption of drug molecules on the NPs surface, resulting in faster dissociation than entrapped drug molecules in the NPs hydrophobic core. However, the HSB‐510 did not show the burst release of the PI3‐Kδ/HDAC6 inhibitor. PI3‐Kδ/HDAC6 inhibitor is comparably more hydrophobic than SAHA and IDL and hence it can easily entrap into the hydrophobic core of the NPs, resulting in it's slow and sustained release.

### 2.5 Cellular uptake study of Rho‐B NPs

**FIGURE 2:**
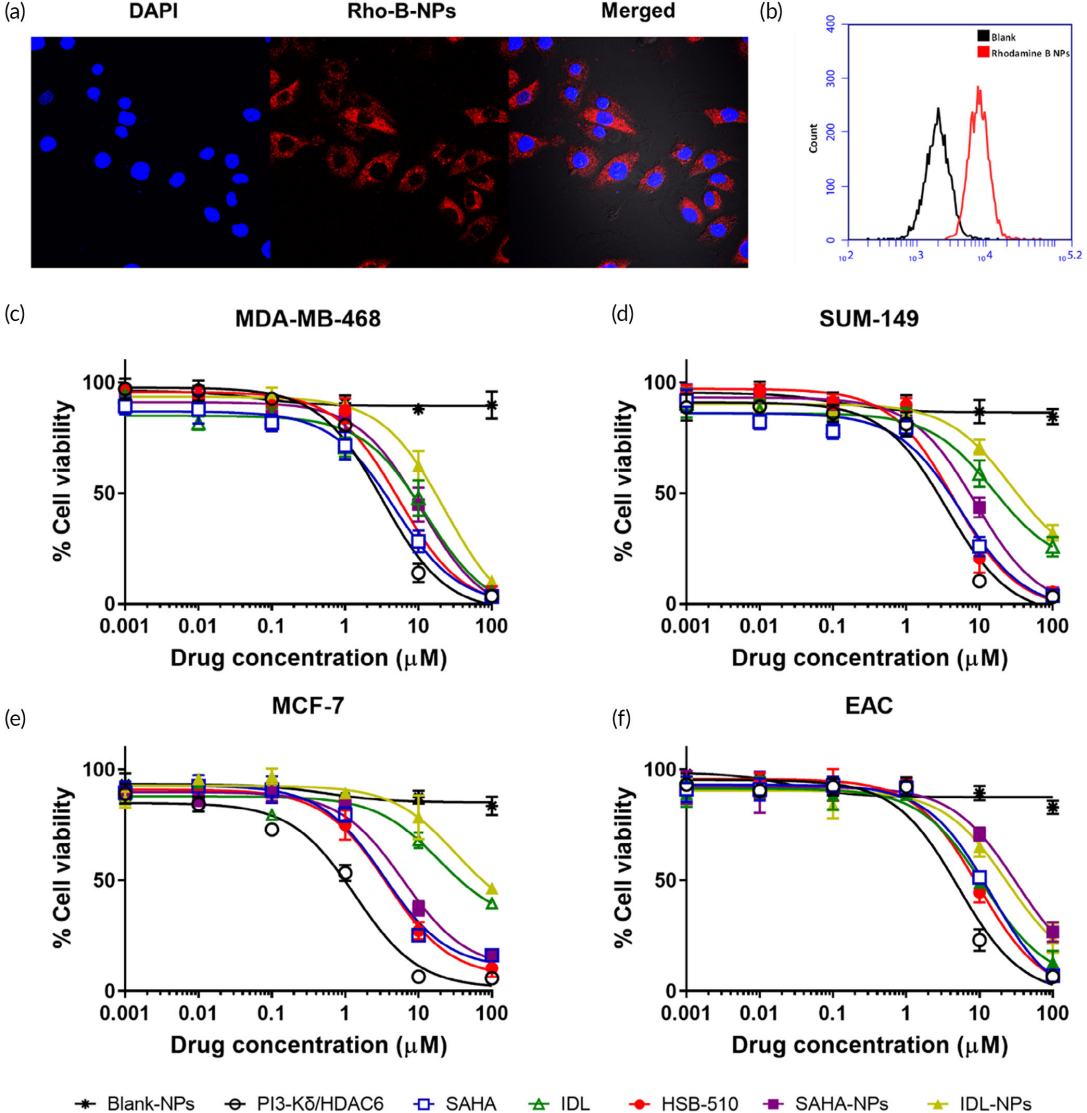
(a) Confocal laser scanning microscopic images of Rho‐B nanoparticles (Rho‐B‐NPs) internalization assessed on MDA‐MB‐468 cell lines. (b) Flow cytometry analysis of Rho‐B‐NPs treated cells (5 h), blank NPs in black, Rho‐B‐NPs in red. (c–f) Percent cell growth inhibition plots of free drug and their nanoformulations.

The CLSM images (Figure 2a) clearly demonstrate the fluorescence of Rho‐B NPs throughout the cytosol, confirming the cellular internalization of Rho‐B NPs. Furthermore, the results indicate that, in contrast to unstained cells, about 96% of cells were positive for Rho‐B staining after 5 h exposure with Rho‐B‐NPs, as determined by FACS analysis (Figure 2b).

### 2.6 Cytotoxicity studies

The cytotoxicity of blank NPs was assessed in healthy cells (HEK‐293) and breast cancer cell lines (MDA‐MB‐468, SUM‐149, MCF‐7, and EAC) using MTT assay. Even higher doses (0.1 mg/mL) of empty NPs (black star, ✶) were found to be nontoxic for these cells (Figure S5 and Figure 2c–f). Afterward, the cytotoxicity of HSB‐510 in breast cancer cell lines was evaluated and compared with the free PI3‐Kδ/HDAC6 inhibitor. Also, the growth inhibition of breast cancer cell lines treated with SAHA and IDL was evaluated for comparison. Breast cancer cells were incubated with different concentrations (1 nM to 100 μM) of free PI3‐Kδ/HDAC6, SAHA, IDL, and their encapsulated NPs (IDL‐NPs, SAHA‐NPs, and HSB‐510), to evaluate the relative cytotoxicities. The data demonstrated that both free and their NPs showed comparable and dose‐dependent inhibition of cell growth in different breast cancer cell lines (Figures 2c–f). After internalization into the cell, NPs were degraded by enzymatic and hydrolytic processes which resulted in the release of payloads.^49^ The released payloads or anticancer agents binds to their target and inhibit cell growth, which leads to cell death. In addition, it was seen that free agents had lower IC_50_ values in comparison to their encapsulated NPs. We attributed the higher IC_50_ values of anticancer agent encapsulated nanoparticles (HSB‐510, SAHA‐NPs, and IDL‐NPs) due to the slower release of anticancer agents from their encapsulated nanoparticles. Free anticancer agents diffuse freely into cancer cells, leads to an immediate inhibitory effect on their targets. In contrast, NP‐encapsulated anticancer agents slowly released from polymeric core upon internalization, causing a delayed inhibitory effect and results in higher IC_50_ values.^55^ In contrast to SAHA and IDL, treatment of cells with the PI3‐Kδ/HDAC6 inhibitor and its NPs (HSB‐510) has been associated with the lowest IC_50_ values in all four breast cancer cell lines tested (Table 2), thereby confirming that dual inhibitor is more potent than HDAC inhibitor (SAHA) and the PI3‐K inhibitor (IDL) in a panel of breast cancer cell lines.

**TABLE 2:** IC_50_ values of free drugs and their encapsulated nanoparticles in breast cancer cell lines.

Cell line	IC_50_ (μM)					
	PI3‐Kδ/HDAC6	HSB‐510	SAHA	SAHA‐NPs	IDL	IDL‐NPs
MDA‐MB‐468	2.81	5.44	4.7	10.95	12.66	23.33
SUM‐149	2.98	4.5	5.6	8.9	15.6	27.17
MCF‐7	1.37	3.17	3.3	6.21	18.9	32.34
EAC	5.17	9.82	14.39	23.58	20.38	35.25

Abbreviations: EAC, Ehrlich ascites carcinoma; HSB‐10, polylactic acid‐methoxy polyethylene glycol; polylactic acid‐polyethylene glycol‐polypropylene glycol‐polyethylene glycol‐polylactic acid; NP, nanoparticles.

### 2.7 Western blot studies

**FIGURE 3:**
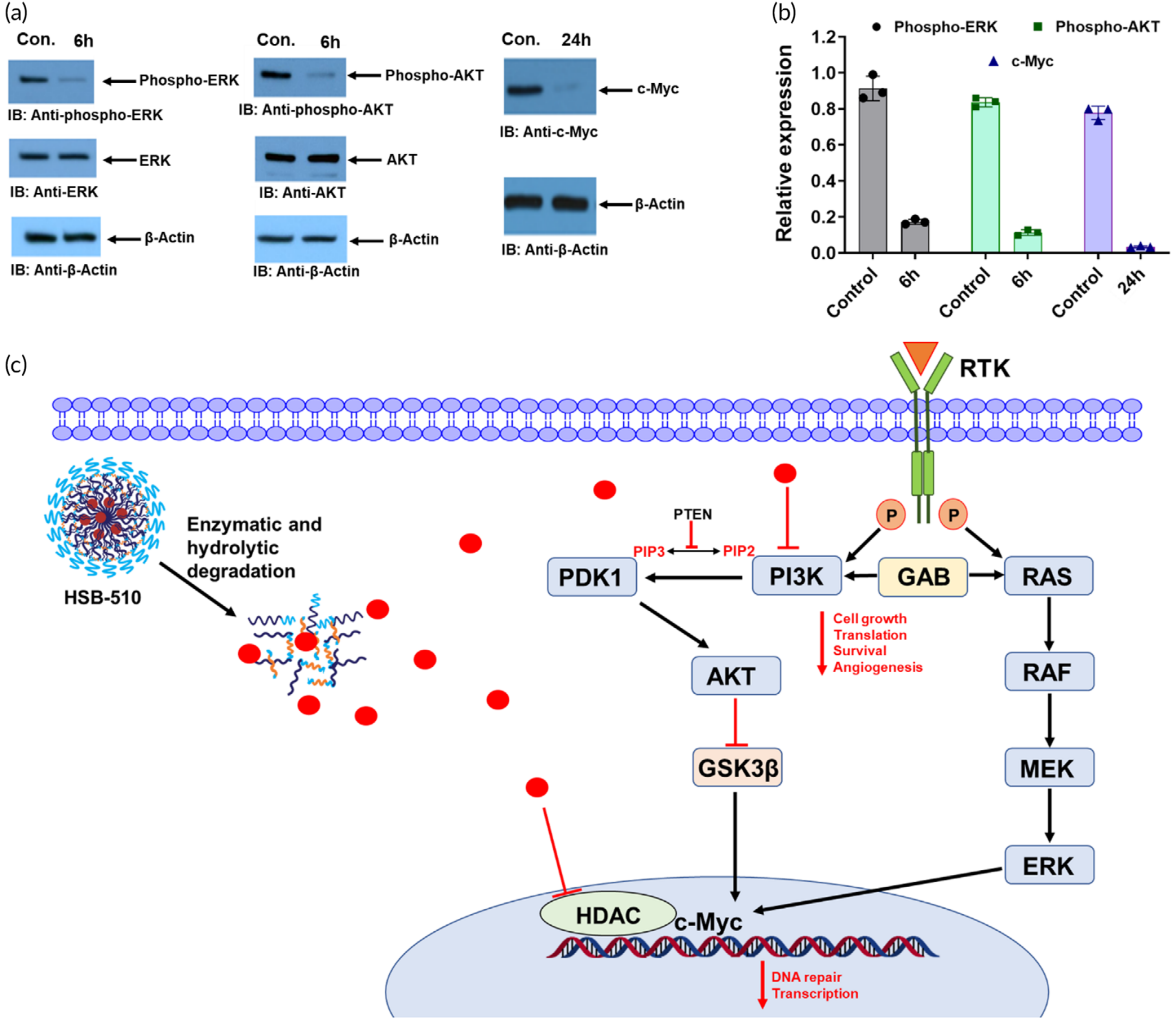
(a) Western blots of SUM‐149 cells, treated with HSB‐510 for 6 h (ERK and AKT) and 24 h (c‐Myc). (b) Bar graph representation of western blot analysis, phospho‐ERK (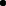), phospho‐AKT (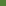), and c‐Myc (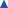), prepared using Image‐J software. (c) Action mechanism of HSB‐510 in cancer cell.

Furthermore, the effects of HSB‐510 on PI3‐K downstream signaling events were assessed. Activation of AKT contributes to cell growth, proliferation, survival, and functions downstream to PI3‐K.^6^ To evaluate the ability of HSB‐510 to inhibit the PI3‐Kδ and suppression of its phosphorylation and activation of downstream kinases involved in cell proliferation and survival, the phosphorylation levels of AKT in SUM‐149 cells after treatment with HSB‐510 were evaluated. The results demonstrated that HSB‐510 treatment in SUM‐149 cells was associated with inhibition of AKT activation, as assessed by detecting phospho AKT levels (Figures 3a,b and S6). Recent studies have shown that there is an intimate interaction between PI3K and RAS pathway via GAB pathway.^56^ Several studies have shown that inhibition of PI3K pathway may result in inhibition of RAF/RAS/ERK/MEK pathway.^57^, ^58^, ^59^ Therefore, activation of ERK was assessed in cells treated with HSB‐510. The results demonstrated that treatment of SUM‐149 cells with HSB‐510 were associated with significant inhibition of phospho‐ERK levels with no change in total ERK (Figure 3a,b and S6), and our results are consistent with published articles.^57^, ^58^, ^59^ Taken together, based on these observations, it was hypothesized that PI3‐Kδ forms a positive feed‐forward loop to enhance oncogenic activity in cancer, making these two proteins suitable for simultaneous targeting to interrupt tumor growth.

c‐Myc is an oncoprotein that is activated in 80% of human cancer types, including breast cancer, which promotes tumorigenesis. More interestingly, there is evidence that overexpression of c‐Myc contributes to drug resistance in breast cancer.^60^, ^61^ Multiple tumors are addicted to c‐Myc, and the reduction in c‐Myc levels leads to tumor regression.^62^ Numerous studies have shown that both HDAC and PI3‐K pathways regulate c‐Myc levels.^31^ Consequently, the effects of HSB‐510 on c‐Myc expression in SUM‐149 cells were evaluated. SUM‐149 cells were treated with HSB‐510, and total lysates from control and treated cells were subjected to immunoblotting with anti‐c‐Myc. The results demonstrated that, in contrast to control, cells treated with HSB‐510 were associated with substantial inhibition of c‐Myc levels (Figures 3a,b and S6). Downregulation of phospho‐ERK, phospho‐AKT, and c‐Myc levels confirms the action mechanism of HSB‐510 in cancer cell line.^31^, ^59^ Together, these results suggested that HSB‐510 has considerable potential to be useful in the clinic as a new inhibitor of c‐Myc for the treatment of breast cancer.

### 2.8 Tumor uptake of ICG and ICG‐NPs

**FIGURE 4:**
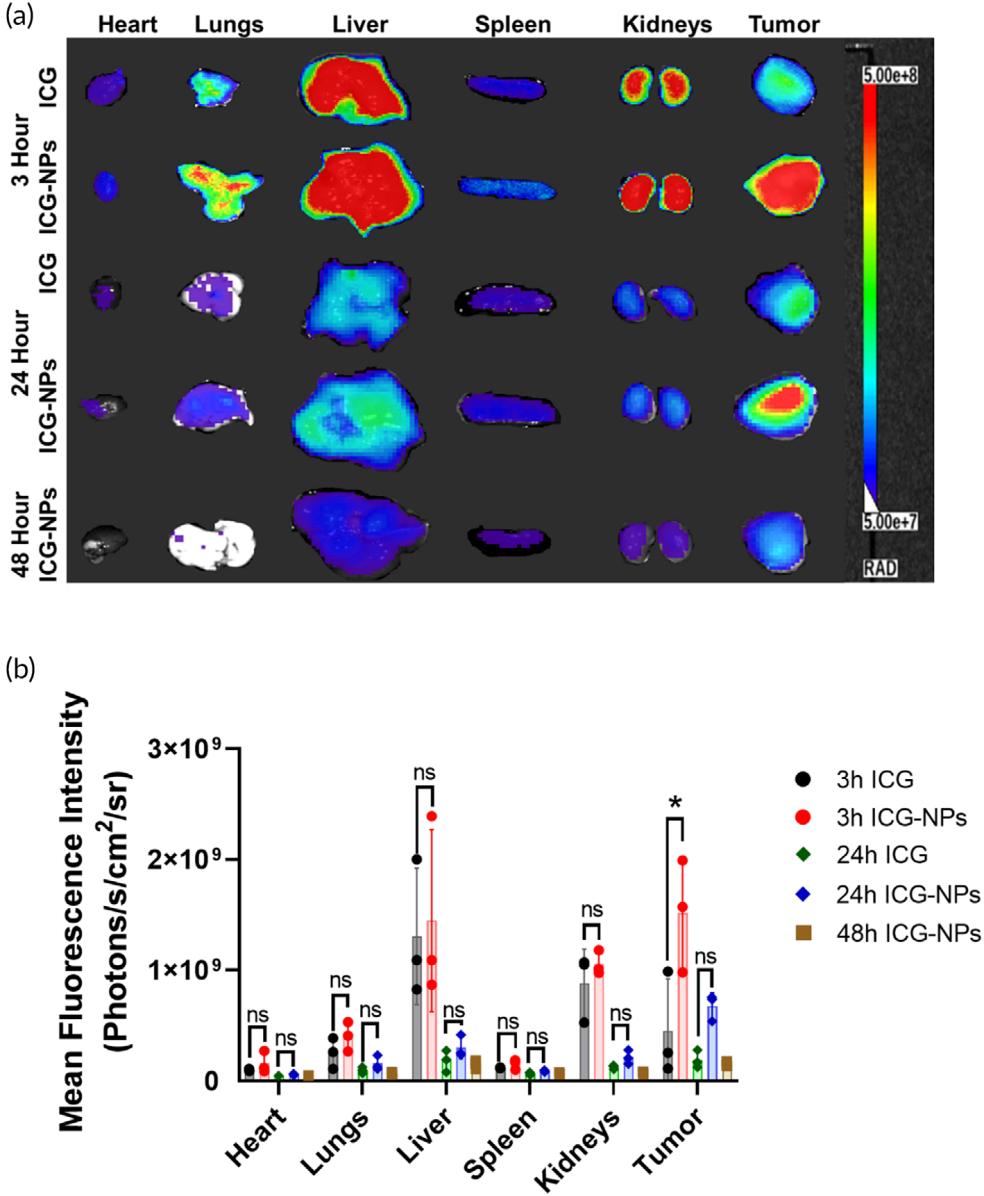
Biodistribution study of indocyanine green (ICG) and ICG nanoparticles (ICG‐NPs). (a) Excised vital organ images of ICG and ICG‐NPs treated mice. (b) Quantitative biodistribution of ICG and ICG‐NPs in vital organs and tumors, taken at different time points, 3 h free (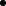), 3 h NPs(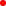), 24 h free (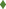), 24 h NPs (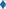), and 48 h NPs (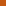). Fluorescence represents the radiant efficiency expressed as (photon/s/cm^2^/steradian).

Biodistribution of ICG and ICG‐NPs was evaluated in EAC tumor‐bearing mice. In contrast to free ICG, a strong fluorescence signal was detected in the tumors of mice treated with ICG‐NPs at 3 h (Figure S7). Free ICG signal decayed rapidly due to faster body clearance, so we did not observe free ICG fluorescence signal at 24 and 48 h time points (data not shown).^63^, ^64^ For a better insight of biodistribution, all animals were sacrificed, and the fluorescence of vital organs was measured ex‐vivo (Figure 4a). At 3 h time point, a substantial difference in tumor uptake was seen when fluorescence signals were compared between free and NPs‐bound ICG (Figure 4a). Moreover, at 24 and 48 h, free ICG is mostly eliminated from the body, while the ICG‐NPs showed the fluorescence signal in the tumor. As the NPs circulation time is longer than the free drug, the accumulation of NPs at the tumor site increases due to its leaky vasculature and poorly developed lymphatic system.^37^, ^54^ Figure 4b shows the quantitative fluorescence signals measured from ex‐vivo image analysis of vital organs, including tumors at all three‐time points. Quantitative fluorescence data of all organs clearly showed the higher accumulation of ICG‐NPs at tumor site in comparison with free ICG, for all time points. Free ICG is majorly distributed in the liver, followed by kidneys, tumor, lungs, and spleen. ICG‐encapsulated polymeric nanoparticles are majorly distributed in tumor followed by liver, kidneys, lungs, and spleen. The ICG‐NPs accumulated at tumor site were significantly higher as compared to free ICG through EPR effect.^36^, ^49^ Accumulation of ICG and ICG‐NPs in liver and spleen could be due to the hepatic biliary excretion and reticuloendothelial system (RES), while the accumulation in lungs could be due to the pulmonary endothelial cells.^65^, ^66^ Accumulation of ICG in kidneys could be due to it's glomerular filtration; however, some reports also confirmed the excretion of nanoparticles (>8 nm) through kidneys.^67^, ^68^ Therefore, the drug‐encapsulated NPs showed better therapeutic efficacy due to their longer body circulation time and accumulation at the tumor site. These data confirmed that the encapsulation of ICG in NPs increases tumor accumulation compared to free ICG following i.v. administration.

### 2.9 In vivo antitumor efficacy of HSB‐510

**FIGURE 5:**
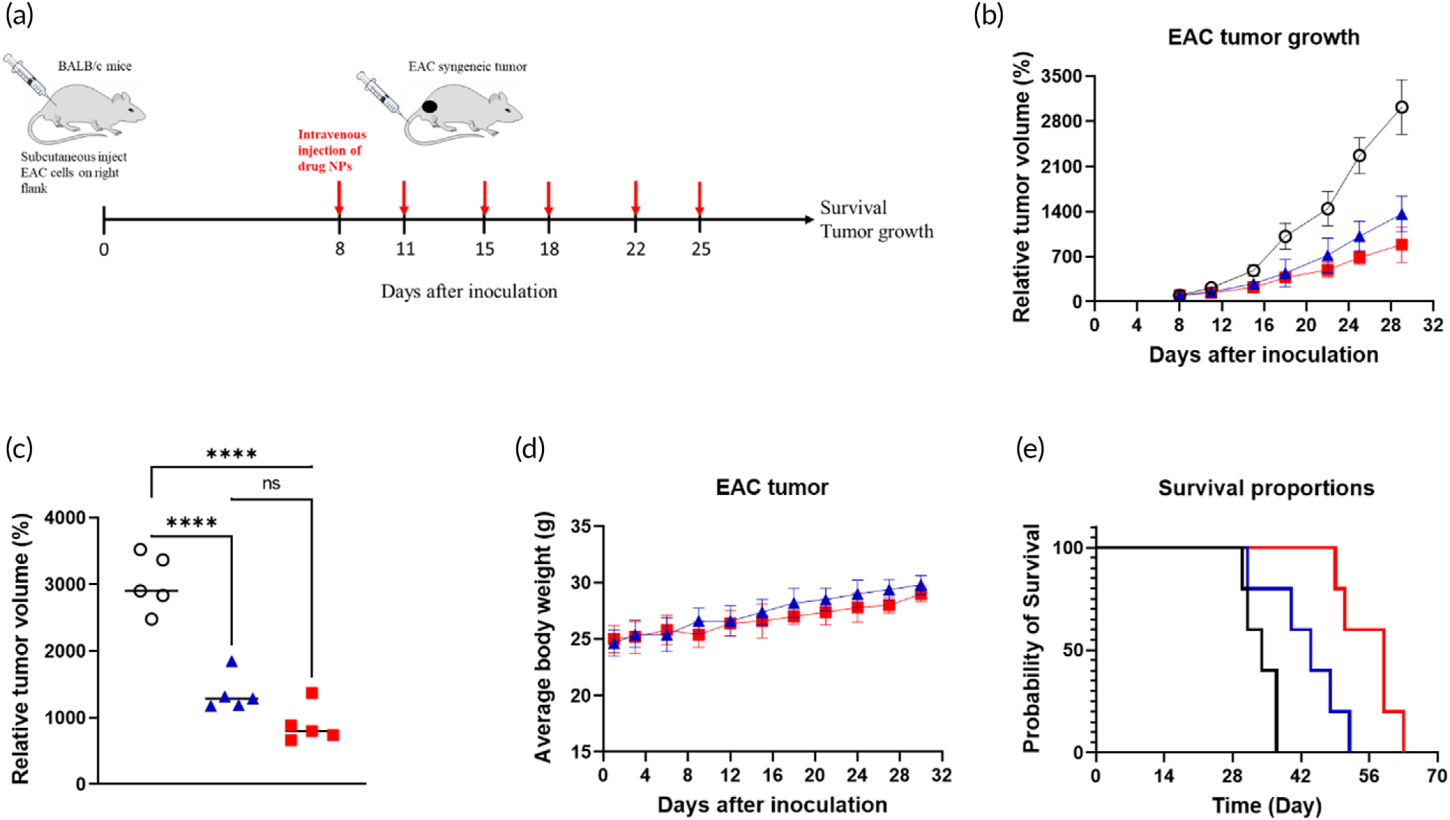
In vivo therapeutic efficacy of HSB‐510 in Ehrlich ascites carcinoma (EAC) syngeneic mice breast cancer model. (a) Treatment schedule. HSB‐510 (12.5 and 25 mg/kg) were intravenously injected into the tail vein at days 8, 11, 15, 18, 22, and 25, after subcutaneous inoculation of EAC cells. (b) Relative tumor volume (%) of EAC‐tumor bearing mice treated with PBS (○), HSB‐510 (*n* = 5) 12.5 (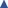) and 25 mg/kg (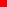). (c) Relative tumor volume (%) of independent mice from each treated group (PBS [○], HSB‐510 12.5 mg/kg [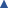] and 25 mg/kg [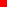]). (d) Variation of body weight over time in EAC‐tumor bearing mice treated with HSB‐510 12.5 mg/kg (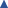) and 25 mg/kg (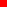). (e) Kaplan‐Meier survival curves of EAC cell line‐derived syngeneic breast cancer tumor bearing mice upon i.v. administration of HSB‐510 12.5 mg/kg (blue), 25 mg/kg (red), and PBS (black). Mice were treated twice a week for 3 weeks.

To investigate whether HSB‐510 is effective in inhibiting the growth of mice tumors, the studies were performed in Balb/c mice bearing established subcutaneous syngeneic Ehrlich breast tumors (EAC). No morbidity and weight loss were observed up to a dose of 25 mg/kg; therefore, we selected doses of 12.5 and 25 mg/kg for tumor regression studies. As compared with the control mice, treatment with 12.5 mg/kg HSB‐510 was associated with partial slowing of Ehrlich tumor growth. By contrast, i.v. dosing of 25 mg/kg HSB‐510 resulted in substantial inhibition of Ehrlich tumor growth, supporting a dose‐response effect (Figure 5a–c). Significantly, there was no overt evidence of toxicity, such as weight loss, associated with HSB‐510 administration.

In the subsequent in vivo study, tumor‐bearing mice were divided into three groups of five mice each, (i) treated with PBS (control), (ii) treated with 12.5 mg/kg HSB‐510, and (iii) 25 mg/kg HSB‐510. The dosing was given intravenously twice a week for 3 weeks, and mice were observed for weight loss and survival for 70 days. The body weight of each mouse in all three groups was not significantly affected, suggesting no major toxicity of HSB‐510 (Figure 5d). The Kaplan–Meier survival curves for each group are shown in Figure 5e. The results demonstrated that the survival of HSB‐510 treated mice (25 mg/kg) was 59 days, which was comparatively improved from the 44 days for HSB‐510 12.5 mg/kg and 29 days for PBS treated group.

**FIGURE 6:**
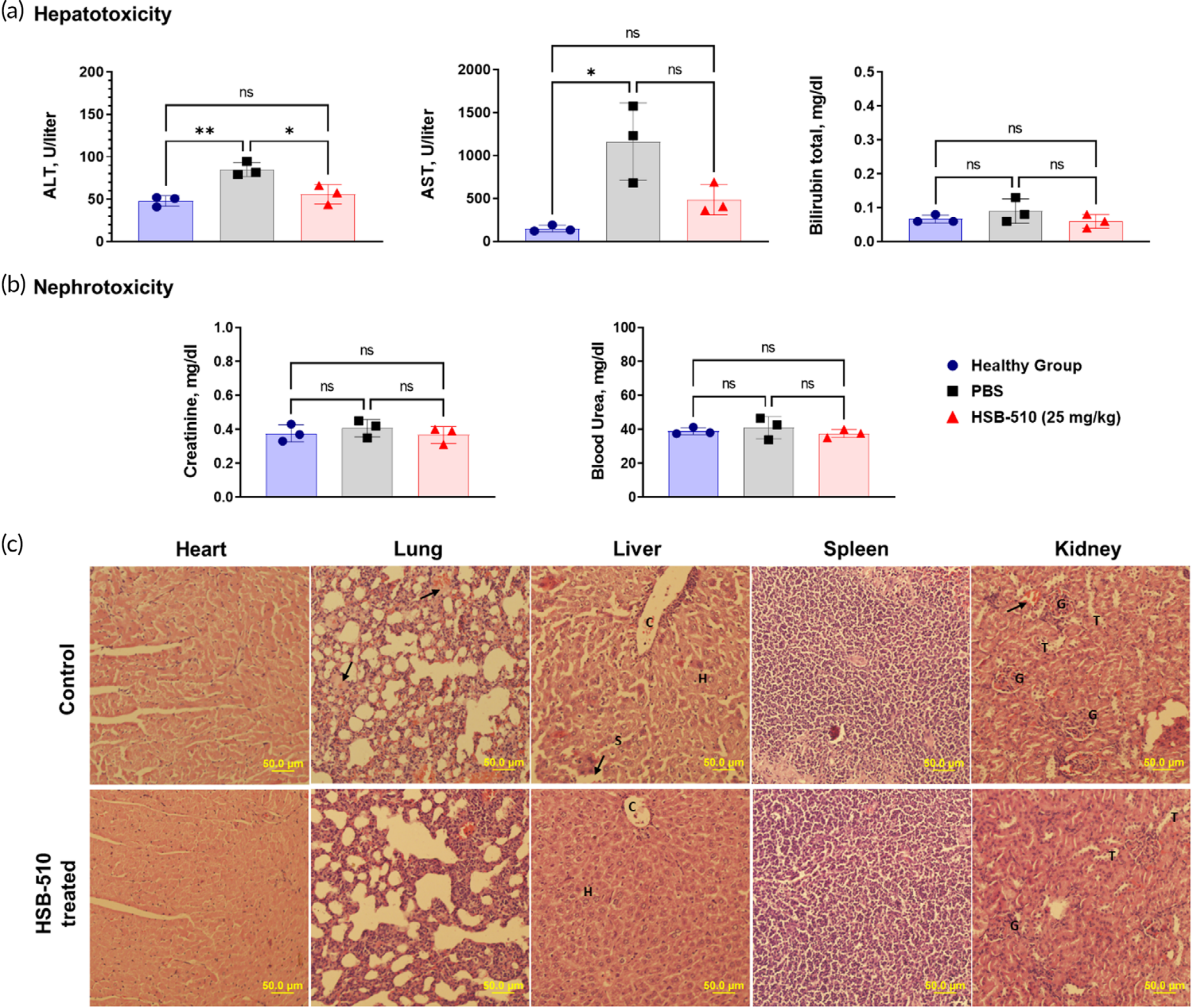
In vivo toxicity studies of HSB‐510 evaluated in Ehrlich ascites carcinoma (EAC) tumor bearing mice. Mice were intravenously administered with 25 mg/kg dose of HSB‐510, twice a week for 3 weeks (total six doses). The blood was collected 48 h after the last dose of HSB‐510 for hepatotoxicity and nephrotoxicity studies. The key organs (heart, lung, liver, spleen, and kidney) were preserved for pathological studies. (a) *Hepatotoxicity studies*: Alanine aminotransferase (ALT), aspartate aminotransferase (AST), and total bilirubin levels were recorded for mice in the healthy group (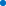), untreated group (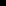), and HSB‐510 treated group (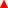) (mice *n* = 3). *(b) Nephrotoxicity studies*: creatinine and blood urea levels were recorded for mice in the healthy group (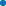), untreated group (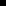), and HSB‐510 treated group (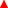) (mice *n* = 3). (c) Hematoxylin and eosin‐stained images of heart, lung, liver, spleen, and kidney sections of HSB‐510 treated and PBS (untreated) mice group. Lung tissue—fibrotic changes (arrows). Liver showing the “S”‐liver sinusoids, “*H*”‐*hepatocytes*: “C”‐congestion in portal veins, vacuolization (arrows). Kidney glomeruli hemorrhage (arrow), G, glomeruli; T, tubules; Scale: 50 μm.

In vivo toxicity of HSB‐510 was studied in EAC tumor‐bearing Balb/c mice. Following 3 weeks of dosing with 25 mg/kg HSB‐510, blood was collected in EDTA tubes and was processed for liver function test (Figure 6a) and kidney function test (Figure 6b). The levels of alanine aminotransferase (ALT), creatinine, blood urea, and bilirubin did not change in comparison to healthy mice. The elevated levels of aspartate aminotransferase (AST) were seen in both PBS‐ and HSB‐510 treated mice. EAC tumor resulted in elevated levels of ALT and AST due to liver necrosis and inflammation.^69^ Treatment with HSB‐510 helped in controlling the ALT and AST levels, confirming the anticancer effect towards EAC tumors.

To get better insight into the in‐vivo toxicity due to the HSB‐510, histopathological studies of vital organs were done in comparison with the untreated mice group using H&E staining (Figure 6c). HSB‐510‐treated mice showed normal heart and spleen. Focal alveolar congestion and fibrotic changes (arrows) were observed in lung tissue sections from the untreated (control) group. Liver tissue of the untreated group showed mild sinusoidal congestion, congestion in portal veins (c), and vacuolization (arrows). While the liver of the HSB‐510‐treated group showed normal hepatocytes and sinusoids. Mild inflammatory response and congestion in the central vein were found in HSB‐510 treated mice liver. Normal renal cortex and glomerular tufts can be seen in HSB‐510 treated kidney tissue section. While the kidney tissue section of the untreated group showed glomeruli hemorrhage (arrow). All these changes suggested that HSB‐510 treatment is effective for cancer therapy without toxicity.

## 3 DISCUSSION

Targeted dual inhibitors have been extensively studied recently for cancer therapy due to their synergistic efficacy and lower toxicities. However, lower water solubility limited their i.v. administration and hence their pharmacological parameters, such as circulating half‐life and bioavailability. For example, CUDC‐907, a dual inhibitor of HDAC/PI3‐K currently in clinical development, necessitated daily dosing of 50–300 mg/kg for effective antitumor activity in tumor models.^31^, ^59^, ^70^ The present study was performed to assess the i.v. delivery and antitumor efficacy of a novel nano‐formulation of PI3‐Kδ/HDAC6 dual inhibitor.

Biodegradable polymeric NPs delivery system is currently becoming a hot area for effective in vivo delivery of anticancer therapeutic agents. In addition, polymeric NPs can be synthesized in such a way that they have their own biological activity to enhance the therapeutic efficacy of encapsulated anticancer agents.^41^, ^50^ Polymeric Pgp/MDR modulators, such as pluronic or poloxamers have been used as drug delivery systems. Pluronic interacts with the cell membrane where drug efflux transporters are localized and change its properties which are critical for the proper functioning of ATP‐binding cassette (ABC) transporters (Pgp, MRP, BCRP).^41^

To the best of our knowledge, none of the nanomedicine formulations based on hybrid polymeric systems have been developed for the preclinical and clinical evaluation for cancer therapy. Moreover, nano‐formulation to deliver dual‐targeted inhibitors is also limited. In this study, pluronic L‐61 was used to synthesize PLA‐based block copolymers (PLA/L‐61_100_, PLA/L‐61_75_, PLA/L‐61_50_, and PLA/L‐61_25_) with different mole ratios of L‐61 and mPEG for a constant mole amount of l‐lactide. These block copolymers were further evaluated to prepare NPs using nanoprecipitation method. However, penta‐block copolymeric NPs are not stable due to hydrophobicity of the block copolymer or unavailability of hydrophilic chains on the outer surface of the NPs. Therefore, another hydrophilic initiator (mPEG) was used with pluronic L‐61 to synthesize PLA‐based block copolymer, a combination of di‐block and penta‐block copolymers (hybrid block copolymers). NPs prepared with PLA/L‐61_25_ were the most stable in compared to other hybrid block copolymers, including PLA/L‐61_100_, PLA/L‐61_75_, and PLA/L‐61_50_. The results indicated that higher hydrophilic content in the NPs was required for the longer stability of the NPs in the aqueous medium. Hence, hybrid block copolymer PLA/L‐61_25_ was found to be the most suitable for the preparation of HSB‐510 nanoformulation. The HSB‐510 nanoformulation was larger in size in comparison to blank NPs due to the encapsulation of PI3‐Kδ/HDAC6 dual inhibitor inside the NP's core. Dual inhibitor entrapment into the core of the NPs also resulted in slow and sustained release of the payload. The slow and steady release of the PI3‐Kδ/HDAC6 dual inhibitor from HSB‐510 led to 2–3 times higher in vitro IC_50_ values as compared to free PI3‐Kδ/HDAC6 dual inhibitor evaluated in breast cancer cell lines. IC_50_ values of HSB‐510 are comparatively lower than SAHA NPs and IDL NPs due to the synergistic inhibition of HDAC6 and PI3‐Kδ. Mechanism of action of the HSB‐510 was further evaluated through western blot analysis in TNBC SUM‐149 cell line. Downregulation of phospho‐AKT and phospho‐ERK within 6 h of HSB‐510 treatment confirmed the inhibitory effect on PI3‐K signaling pathways, which is the most frequently activated intracellular pathway that contributes to tumor progression. Inhibition of HDACs, regulates the activity or expression of many cellular proteins, including E2F, p53, NFkB, HIF‐1a, STAT‐3, HSP‐90, and c‐Myc.^11^, ^71^ Downregulation of c‐Myc protein was also found in SUM‐149 cells after HSB‐510 treatment. These findings confirmed the simultaneous inhibition of PI3‐K and HDAC signaling pathways. Therapeutic efficacy of most of the drug molecules is limited due to their short half‐life, fast kidney excretion, and lower tumor accumulation. These limitations can be overcome by using polymeric nanoparticle‐based drug formulation, which can accumulate in tumor due to EPR effect.^37^ ICG‐NPs showed higher fluorescence at the tumor site compared to free ICG at each time point (3, 24, and 48 h). This data confirms the improved in vivo tumor uptake, slower and sustained release profile of ICG‐NPs. The in vivo therapeutic efficacy results showed substantial tumor inhibition with HSB‐510. The HSB‐510 inhibited tumor growth by approximately 76% in EAC syngeneic tumor model and it was a dose‐dependent effect.

## 4 CONCLUSION

In summary, we report a novel biodegradable polymeric nanoformulation of PI3‐Kδ/HDAC6 dual inhibitor (HSB‐510) based on PLA block copolymeric system for breast cancer therapy. In vitro studies demonstrate slow and sustained release of the encapsulated PI3‐Kδ/HDAC6 dual inhibitor. The mechanism of cancer suppression is validated by the downregulation of phospho‐ERK, phospho‐AKT, and c‐Myc. In vivo NPs distribution study confirmed the improved tumor accumulation of ICG‐NPs in comparison to free ICG. The in vivo syngeneic mouse model data demonstrates that the NP formulation of PI3‐Kδ/HDAC6 dual inhibitor (HSB‐510) is very active without any toxicity. To the best of our knowledge, this is the first study to demonstrate the use of polymeric nanoformulation of PI3‐Kδ/HDAC6 dual inhibitor (HSB‐510) to improve the efficacy and safety of dual inhibitor molecules for cancer therapy. The nanoparticle system presented here provides a new and better polymeric formulation to deliver chemo drugs at tumor sites. The HSB‐510 has proven its anticancer therapeutic efficacy in breast cancer and can be tested on other tumor indications.

## 5 MATERIALS AND METHODS

Please see Supplementary Information for detailed information on materials.

### 5.1 Cell lines

Breast cancer cell lines MDA‐MB‐468 (TNBC), SUM‐149 (TNBC), and MCF‐7 (ER+ and PR+) were received as a gift from Dana Farber Cancer Institute, Harvard Medical School, Boston, USA. Murine breast cancer EAC cell line (ER+) was procured from National Centre for Cell Science, India.

### 5.2 PLA‐based hybrid block copolymer synthesis and characterization

The procedure of PLA‐based hybrid block copolymer (comprising penta‐block and di‐block) synthesis is detailed in Supplementary Information.

### 5.3 NP preparation and characterization

NPs were prepared using the nanoprecipitation method.^52^, ^53^ Detailed procedure is given in Supplementary Information. The drug encapsulation efficiency percent (EE%) in nanoparticles was calculated using the following equation:

$$\text{Encapsulation}\;\text{Efficiency}\;\%=\frac{\left(\text{Drug}\right)\text{Total}-\left(\text{Drug}\right)\text{Filtrate}}{\left(\text{Drug}\right)\text{Total}}\times 100$$

Loading capacity percentage (LC%) was calculated by the amount of total entrapped drug divided by the total weight of nanoparticles.

### 5.4 In vitro release studies

In vitro release of dual PI3‐Kδ/HDAC6 inhibitor was done in phosphate buffer saline (PBS, pH ‐7.4). Lyophilized HSB‐510 (20 mg) were suspended in PBS (5 mL) and kept in an incubator shaker at 37°C and 150 rpm. The NPs solution was filtered using Amicon 3 KDa at predetermined time points (every 24 h for 12 days). The collected filtrates were analyzed using the HPLC method.^33^ In the same way, the release of SAHA and IDL was assessed from SAHA and IDL encapsulated NPs.

### 5.5 In vitro studies

#### 5.5.1 Cellular uptake studies of Rho‐B NPs

Details of the assay are given in Supplementary Information.

#### 5.5.2 Cytotoxicity assay

Cytotoxicity of free PI3‐Kδ/HDAC6, HSB‐510, SAHA, SAHA‐NPs, IDL, and IDL‐NPs were evaluated using MTT assay.^44^ Details of the assay are given in Supplementary Information.

#### 5.5.3 Western blot studies

Details of the assay are given in Supplementary Information.

### 5.6 In vivo studies

#### 5.6.1 Animals

Female Balb/c mice of 8–10 weeks of age (23–26 g) were used for all in vivo experiments under ethical clearance (122/IAEC/2019) obtained from Committee for the Purpose of Control and Supervision of Experiments on Animals (CPCSEA), All India Institute of Medical Sciences (AIIMS) New Delhi, India. The animals were maintained under controlled ambient temperature (25 ± 1°C), humidity (55 ± 5%), and 12 h light/12 h dark cycle. To generate tumors, EAC cells (10^8^ cells/100 μL PBS/mouse) were subcutaneously injected into the right hind limb of the female Balb/c mice.^72^ Tumor volume was measured every 2 days using digital Vernier Caliper. Randomization of the mice was done according to tumor size.

#### 5.6.2 In vivo and ex‐vivo biodistribution studies of ICG and ICG‐NPs

Time‐dependent tumor uptake studies were evaluated using EAC syngeneic tumor‐bearing female Balb/c mice to confirm the enhancement of tumor uptake of ICG by NPs formulation. Mice bearing tumors (350–450 mm^3^) were divided into five groups (3 mice in each group). Details of the study are given in Supplementary Information.

#### 5.6.3 Tumor growth inhibition and toxicity studies with HSB‐510

Mice bearing tumors (140–170 mm^3^) were divided into three groups (5 mice in each group). HSB‐510 (12.5 and 25 mg/kg) and PBS (control group) were injected intravenously (injection volume 100 μL) in the lateral tail vein, twice a week for 3 weeks. The mice were not treated with free PI3‐Kδ/HDAC6 dual inhibitor due to its insolubility in PBS. Detailed information about the tumor and body weight measurement, tumor regression calculation, and toxicity studies (histopathology, KFT, and LFT) are given in Supplementary Information.

### 5.7 Statistical analysis

All data is represented as mean ±SD. GraphPad Prism 9 software was used for all statistical analysis. The normality of the data was tested using Shapiro–Wilk test. The statistical significance was determined using ANOVA followed by the Bonferroni test with *p* < 0.05 as the minimal significance level. Significance level: ****p < 0.0001; ***p < 0.001; **p < 0.01; *p < 0.05; ns, not significant.

## AUTHOR CONTRIBUTIONS

**Sachchidanand Tiwari:** Conceptualization (lead); data curation (lead); formal analysis (lead); investigation (lead); methodology (lead); software (lead); writing – original draft (lead); writing – review and editing (lead). **Suiyang Liu:** Data curation (supporting); formal analysis (supporting). **Anees Mohd:** Data curation (supporting); formal analysis (supporting). **Neha Mehrotra:** Formal analysis (supporting). **Ashish Thakur:** Data curation (supporting). **Gregory J. Tawa:** Data curation (supporting); writing – review and editing (supporting). **Gurmit Grewal:** Conceptualization (supporting); writing – review and editing (supporting). **Richard Stone:** Conceptualization (supporting); writing – review and editing (supporting). **Surender Kharbanda:** Conceptualization (equal); formal analysis (equal); funding acquisition (equal); methodology (equal); project administration (equal); resources (lead); supervision (equal); validation (equal); writing – review and editing (equal). **Harpal Singh:** Conceptualization (equal); funding acquisition (equal); methodology (equal); supervision (equal); validation (equal); writing – review and editing (equal).

## CONFLICT OF INTEREST STATEMENT

Harpal Singh and Surender Kharbanda are members of the Scientific Advisory Board of Hillstream BioPharma Inc. Surender Kharbanda is an equity holder in Hillstream BioPharma. Harpal Singh is a consultant to Nanogen Pvt Ltd., New Delhi, India. Other authors have no potential conflicts of interest.

### PEER REVIEW

The peer review history for this article is available at https://www.webofscience.com/api/gateway/wos/peer-review/10.1002/btm2.10541.

## Supporting information


**Data S1:** Supporting Information

## Data Availability

The data that support the findings of this study are available on request from the corresponding author. The data are not publicly available due to privacy or ethical restrictions.
