# Supplementary material for: Quatramer™ encapsulation of dual‐targeted PI3‐Kδ/HDAC6 inhibitor, HSB‐510, suppresses growth of breast cancer
Source: Bioeng Transl Med. 2023 May 12;8(5):e10541. doi: 10.1002/btm2.10541 (PMC10487321; doi:10.1002/btm2.10541)
Supplement: Supplementary file 1 — Data S1: Supporting Information [file BTM2-8-e10541-s001.docx]

# Quatramer^TM^ encapsulation of Dual-targeted PI3-Kδ/HDAC6 Inhibitor, HSB-510, Suppresses Growth of Breast Cancer

Sachchidanand Tiwari1, Suiyang Liu2, Mohd Anees1, Neha Mehrotra1, Ashish Thakur3, Gregory J. Tawa3, Gurmit Grewal3, Richard Stone2, Surender Kharbanda2, and Harpal Singh1,4

1Centre for Biomedical Engineering, Indian institute of Technology Delhi, New Delhi 110016, INDIA

and

2Dana Farber Cancer Institute, Harvard Medical School, Boston 02115, MA, USA

and

3National Center for Advancing Translational Sciences, National Institutes of Health, 9800 Medical Center Drive, Rockville,

Maryland 20850, USA and

4Department of Biomedical Engineering, All India Institute of Medical Sciences Delhi, New Delhi 110029, INDIA

# Supplementary Section

**Materials and methods Materials**

L-Lactide was procured from Corbion (Amsterdam, Netherlands). Methoxy polyethylene glycol (Mn-5000), L-61 poloxamer (PEG-PPG- PEG; Mn-2000), stannous octoate and Rhodamine-B (Rho-B) were procured from Sigma Aldrich (India). Single agent PI3-Kδ/HDAC6 dual inhibitor (Fig. S1) was in housed synthesized as reported by Thakur et al.^1^. Chemical name and structure are given in Fig. S1. Vorinostat (SAHA) and Idelalisib (IDL) were purchased from Medchem express (USA). Indocyanine green (ICG) was procured from Aurolab, India. MTT was purchased from HiMedia (India). Cell culture media (DMEM), fetal bovine serum (FBS), and pen/strep solution were procured from Gibco (ThermoFisher Scientific, India).

# PLA block copolymer synthesis and characterization

PLA based hybrid block copolymers comprising penta-block and di- block, were synthesized through ring-opening polymerization (ROP). In brief, monomer (L-lactide) and initiator (L-61) or a mixture of initiators (L-61+ mPEG) were added into a vacuum dried two-neck round bottom flask and heated with continuous stirring under nitrogen environment to get well mixed molten phase. At 80°C, stannous octoate (0.005 wt % of L-lactide) was added into the flask, and the mixture was heated to 170°C for three hours with continuous stirring under a nitrogen environment. After three-hour, the flask was cooled to room temperature, and dichloromethane (DCM) was added to dissolve the solid content. Methanol (10X volume of DCM) was used to precipitate the block copolymers and solubilize the unreacted monomer, initiator, and catalyst. The precipitated polymer was filtered and dried overnight under a vacuum. Gel permeation

chromatography (GPC, Viscotek GPC system, Malvern Panalytical, United Kingdom) was performed to study the molecular weight and poly dispersity index (PDI) of synthesized polymers at room temperature with tetrahydrofuran as the mobile phase. 1H NMR of synthesized polymers were done in CDCl3 at 400 Hz (Bruker, USA).

# Nanoparticles preparation and characterization

To evaluate the suitable PLA based hybrid block copolymer for in-vitro and in-vivo studies, blank NPs from all synthesized block copolymers were prepared separately using the nanoprecipitation method. In brief, PLA block copolymer (100 mg) was dissolved in 5 ml acetonitrile at 50°C for 10-15 minutes. After complete dissolution, PLA block copolymer solution was injected in an aqueous F127 emulsifier solution (5 mg/ml) during continuous stirring (600-700 rpm) using a 26-gauge needle. NPs solution was kept on stirring at room temperature to evaporate acetonitrile and stabilize nanoparticles. Suitable block copolymer was evaluated through NPs stability study. NP stability was defined according to their change in size compared to the initial size measured with the DLS instrument (Anton Paar Lightsizer500, GmbH, Austria). For this study, 10 mg/ml NPs in double distilled water were kept at 4°C and size measurements were done on daily basis up to 15 days.

The selected block copolymer (PLA/L6125) was then used to prepare dual inhibitor encapsulated NPs (HSB-510). For NPs preparation, dual PI3-Kδ/HDAC6 inhibitor was dissolved in DMSO (1 mg/30 µL) and mixed with PLA block-copolymer solution (1:10, weight by weight ratio of drug and polymer). This complete solution was injected into F-127 emulsifier solution to prepare NPs. These nanoparticles were filtered through Amicon 3kDa ultrafilter (Millipore) using centrifugation at 4000 rpm for 40 minutes to remove unencapsulated dual PI3-Kδ/HDAC6 inhibitor. Encapsulated NPs were then lyophilized and stored at -20°C until use. The filtrate was collected and analyzed for unencapsulated dual PI3-

Kδ/HDAC6 inhibitor using HPLC methods reported elsewhere (1). The above method was also used to prepare SAHA, IDL, Rho-B and ICG encapsulated NPs. Nanoparticles size and zeta potential were assessed using DLS instrument. Shape, size, and morphology of the NPs were also characterized with high-resolution transmission electron microscopy (HR-TEM, FEI Tecnai TF20, USA).

**Cellular uptake studies of Rho-B NPs**

To confirm the cellular uptake of Rho-B NPs, MDA-MB-468 cells were seeded into a six-well plate containing gelatin-coated coverslip ^2^. After 70-75% confluency, cells were then treated with Rhodamine-B encapsulated NPs for 5 hours. Cells were washed with PBS twice and stained with DAPI in culture medium for 10 minutes in the dark. After incubation, coverslips were washed with PBS three times, removed from the six-well plates, and placed on a glass slide using a 15 µl glycerol:PBS (1:1, v/v) solution. These cells were then imaged under a confocal laser scanning microscope (CLSM FV1000; Olympus, Japan). The cellular uptake of Rho-B NPs was also assessed using flow cytometry (FACS-can, Becton Dickson, USA) after 5 hours.

# Cell culture and cytotoxicity assay

Breast cancer cell lines including MDA-MB-468, SUM-149, MCF-7 and EAC were grown in DMEM medium containing 10% FBS and 100 ug/ml pen/strep solution. Cells were then plated in flat bottom 96 well plates (Corning) in cell density of 2500-5000 cells per well. After overnight incubation, cells were treated with different concentrations of free PI3-Kδ/HDAC6, HSB-510, SAHA, SAHA NPs, IDL, and IDL NPs for 72 h. After 72-hour incubation, cells were treated with 10 µl MTT solution (5 mg/ml in PBS) and incubated for 4 hours in CO2 incubator. The medium was removed, and 100 µl DMSO was added to dissolve formazan crystals. The absorbance of each well was then measured at 570 nm using a microplate reader (Bio-Tek, USA). The IC50 values were determined using GraphPad Prism 9 software. To evaluate the PLA based block copolymer related toxicities on healthy cells, the HEK-293 cells (5000 cells/well in 96 well plate) were treated with different concentrations of blank NPs for 72 hours. All the experiments were done in triplicates.

**Western blot studies**

Cells were harvested and rinsed with ice-cold phosphate buffered saline (PBS). Ice-cold lysis buffer [0.5 ml; 20 mM Tris (pH7.5), 150 mM NaCl, 1 mM EDTA, 1% Triton X-100, 2.5 mM sodium pyrophosphate, 1 mM β-glycerophosphate, 1 mM Na3VO4, 1 mg/ml leupeptin,1 mM PMSF] was added to 1 x 10^7^ cells and sonicated on ice four times for 5 seconds each followed by microcentrifugation for 10 minutes at 4^o^C. The lysates were subjected to immunoblotting with anti-β-Actin (Cell Signaling Technology), anti-p-AKT S473, anti-AKT, anti-p-ERK, anti-ERK and anti-c-Myc antibodies (Cell Signaling Technology). Antigen-antibody complexes were visualized by enhanced chemiluminescence (GE Healthcare, USA).

**In-vivo and ex-vivo biodistribution studies of ICG and ICG NPs**

Time-dependent tumor uptake studies were evaluated using EAC syngeneic tumor model to confirm the enhancement of tumor uptake of ICG by NPs formulation. Bio-distribution of free and NP encapsulated ICG was analyzed by whole-body fluorescence imaging as well as ex-vivo imaging of main body organs i.e., heart, lung, liver, spleen, kidney, and tumor. Mice bearing tumor (350-450 mm^3^) were divided into five groups (3 mice each group; Group 1 and 2- Free ICG, Group 3,4 and 5- ICG-NPs). ICG and ICG NPs (1 mg/kg dose of ICG) were injected into mice via tail vein (100 μL) injection. At 3h, 24h and 48h after injection of ICG dye and ICG NPs, the mice were anesthetized using Ketamine Xylazine injection (IP) and imaged using in vivo imaging system (IVIS Lumina XR, Perkin Elmer, USA). After in vivo imaging, mice were sacrificed, and their vital organs and tumors were excised and examined using IVIS to get ex-vivo distribution of ICG dye and ICG-NPs. The fluorescence from each organ was analyzed by the Aura image software.

# Tumor growth inhibition and toxicity studies

Tumor volume was measured using a digital Vernier Caliper and calculated using the following equation:

Tumor volume (mm^3^) = (W^2^ × L)/2

where W and L are width and length of the tumor, respectively.

The tumor and body weight were measured every third day through a digital Vernier Caliper and digital weighing balance. Tumor regression was calculated using the following equation:

$Tumor regression \%=\frac{\Delta C-\Delta T}{\Delta C} X 100$%

Where, **Δ C** = mean tumor volume of the control group on study day of interest – mean tumor volume of the control group on the initial day of dosing and **Δ T** = mean tumor volume of the drug treated group on study day of interest – mean tumor volume of the drug-treated group on the initial day of dosing.

After complete dosing, one mouse each from the control group PBS) and treated (HSB-510, 25 mg/kg) were sacrificed by cervical dislocation. Their vital organs such as heart, lung, liver, kidney, and spleen were harvested and fixed with 4% formalin. The fixed organs were then embedded in paraffin wax and stained with hematoxylin and eosin (H&E) to evaluate the toxicity of the HSB-510 treated mice compared with the untreated group. Images were observed and captured by CLSM, Olympus. Blood (0.5 to 0.7 ml) was collected from the HSB-510 group, control group, and healthy group (n=3) for liver function test (LFT) and kidney function test (KFT).

# Supplemental Figure Legends

**Figure S1:** PI3-Kδ/HDAC6 dual inhibitor structure and chemical name

**Figure S2:** GPC chromatograms of synthesized block

copolymers

**Figure S3A:** ^1^H NMR of PLA/L61100

**Figure S3B:** ^1^H NMR of PLA/L6175 **Figure S3C:** ^1^H NMR of PLA/L6150Figure **Figure S3D:** ^1^H NMR of PLA/L6125 **Figure S4A:** Release profile of SAHA **Figure S4B:** Release profile of IDL

**Figure S5:** Cytotoxicity of blank NPs on HEK-293 cells

**Figure S6:** Replicate image of western blots

**Figure S7:** In-vivo distribution of free ICG and ICG NPs at 3h time point

**Figure S1**. PI3-Kδ/HDAC6 dual inhibitor structure


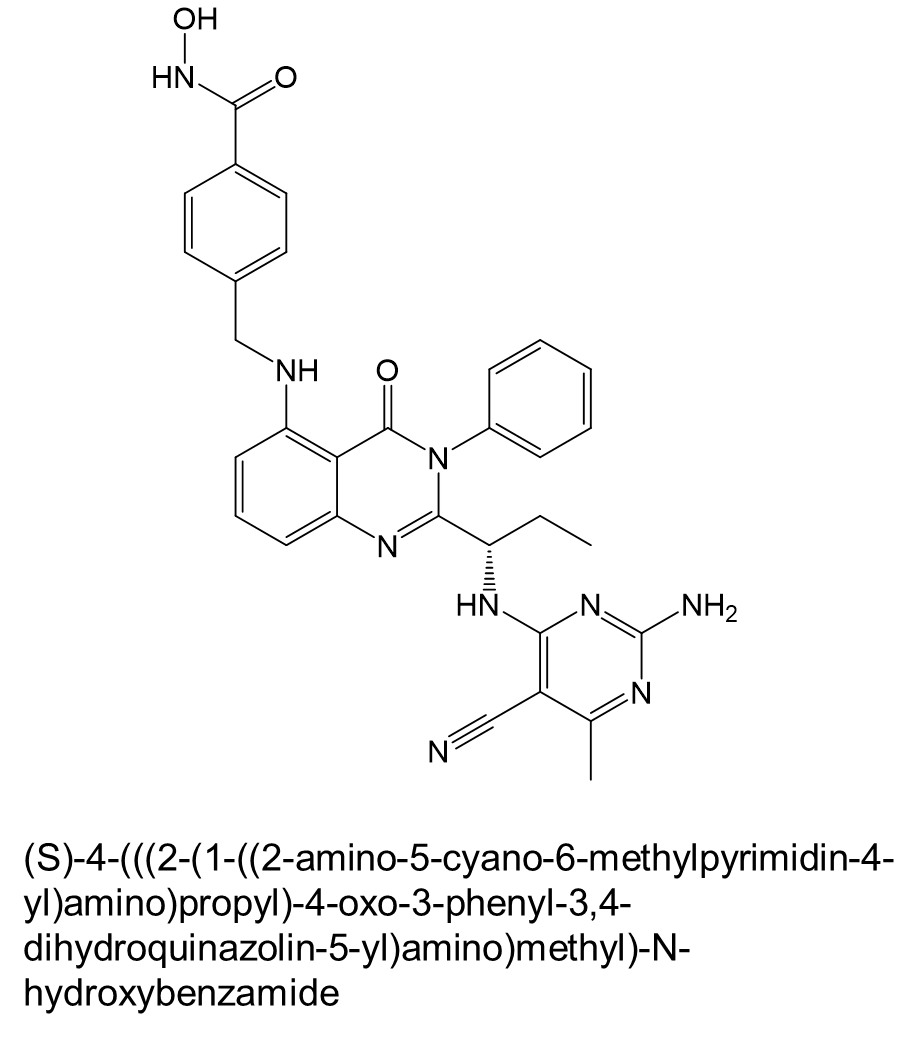


**Figure S2:** GPC chromatograms of synthesized block copolymers


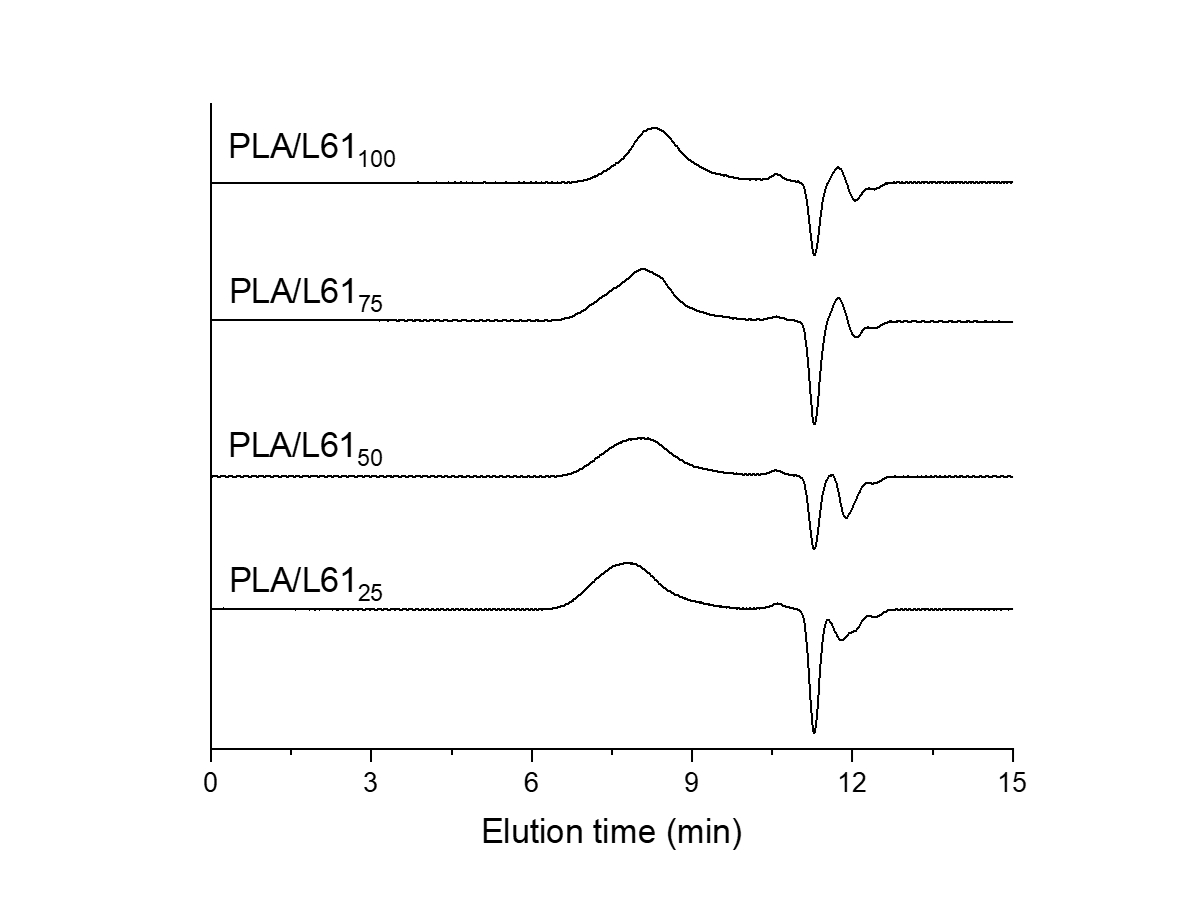


**Figure S3:**

**A.** ^1^H NMR of PLA/L61100


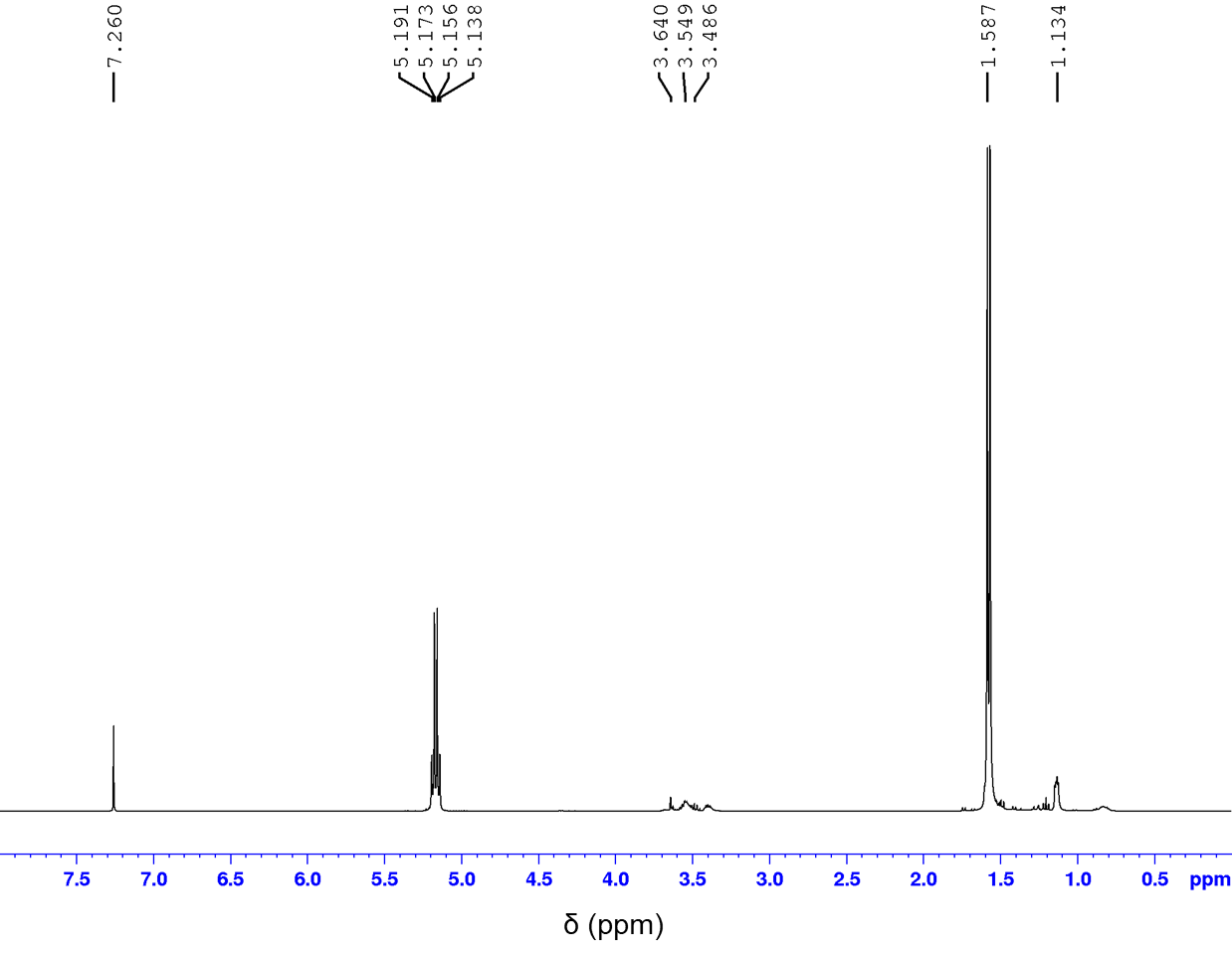


**B.** ^1^H NMR of PLA/L6175


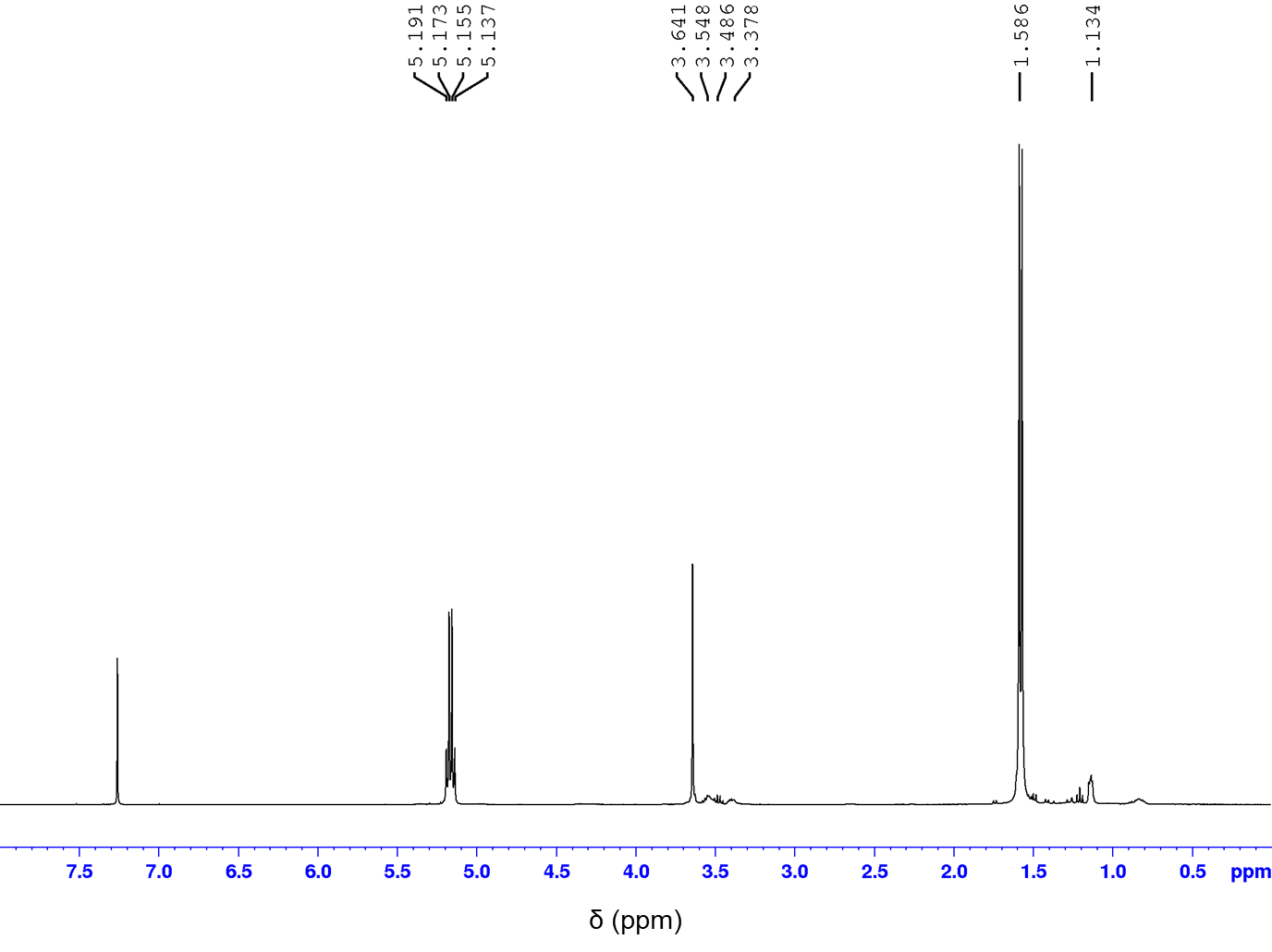


**Figure S3:**

**C.** ^1^H NMR of PLA/L6150


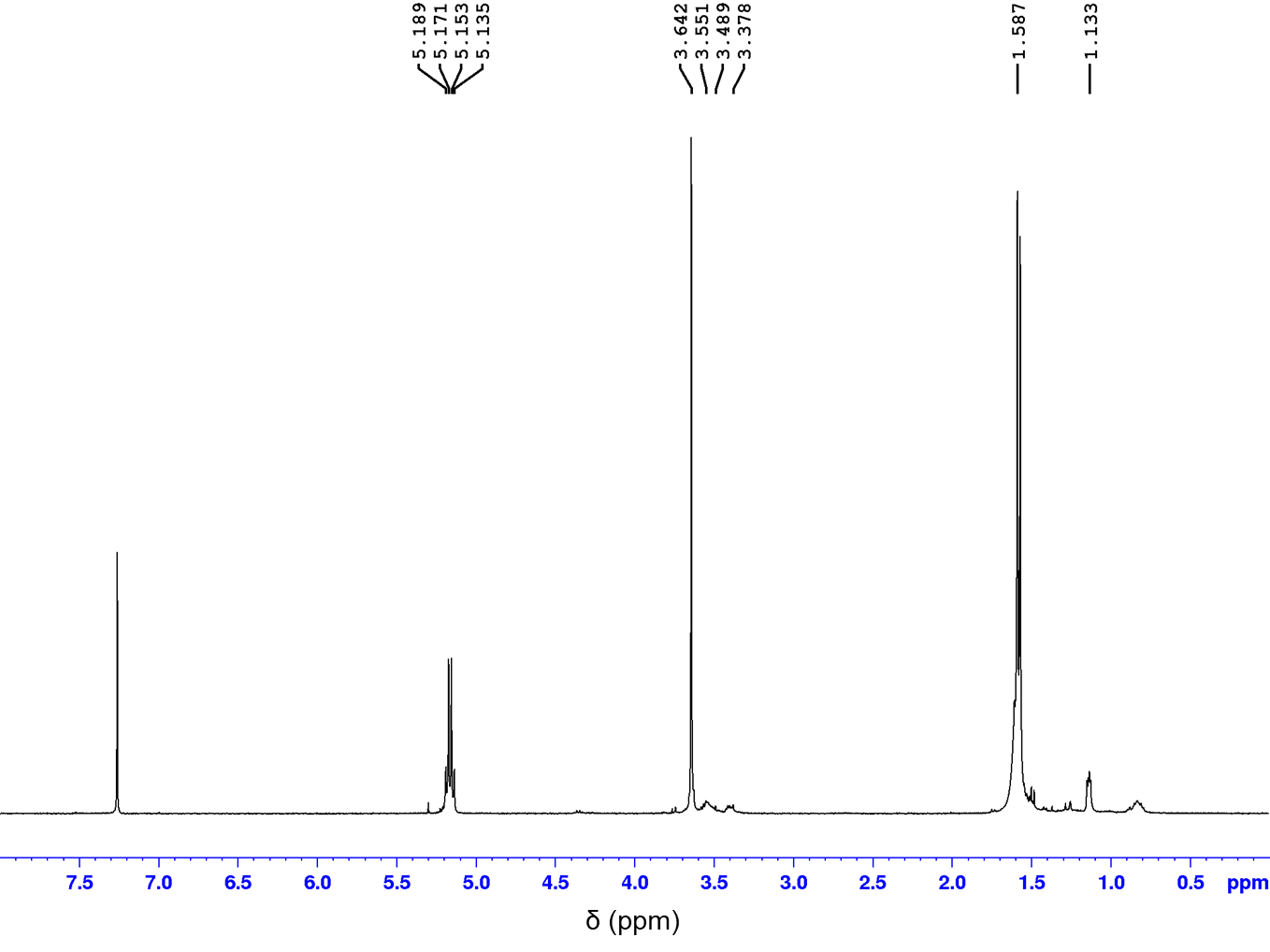


**D.** ^1^H NMR of PLA/L6125


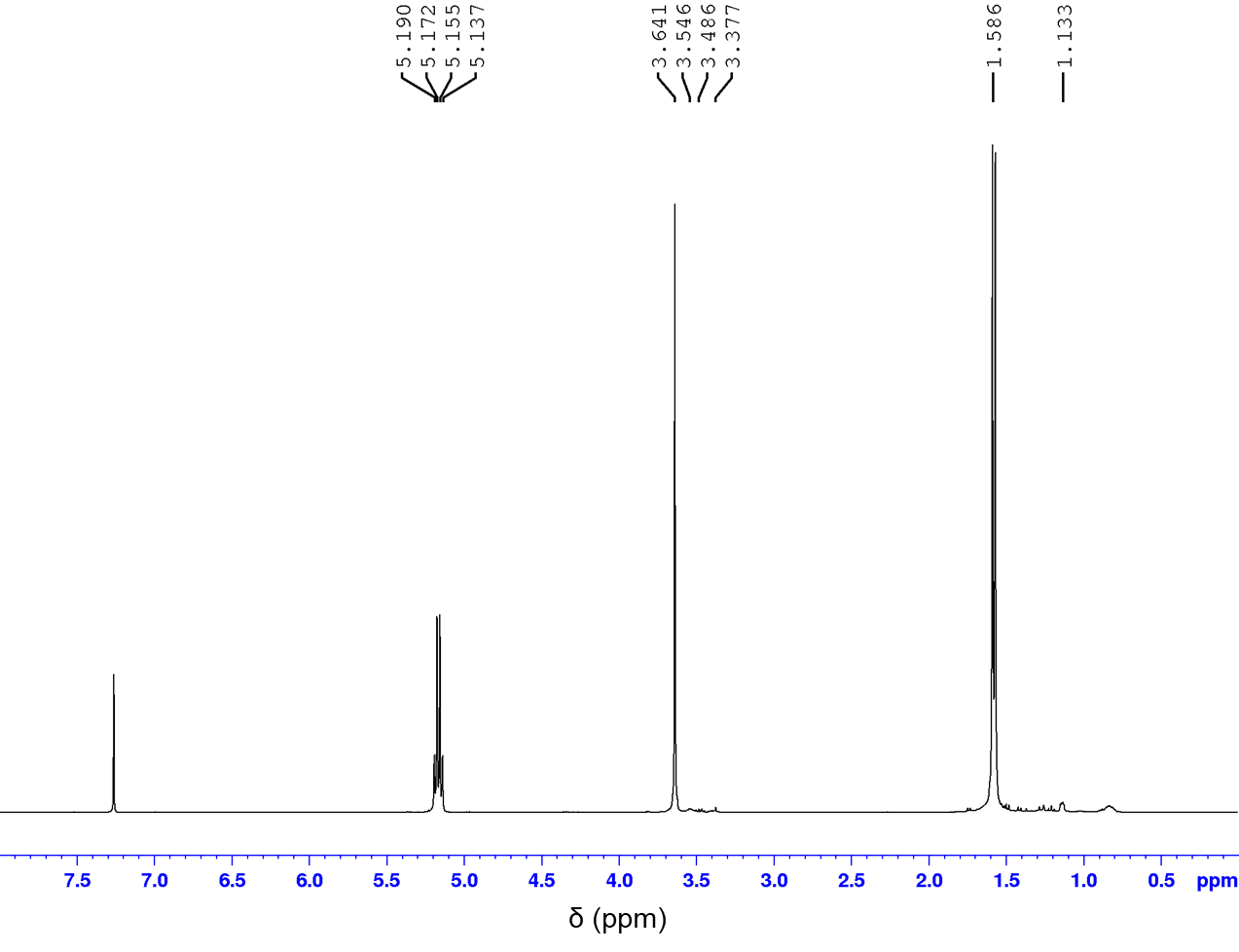


**Figure S4:**

1. Release profile of SAHA.


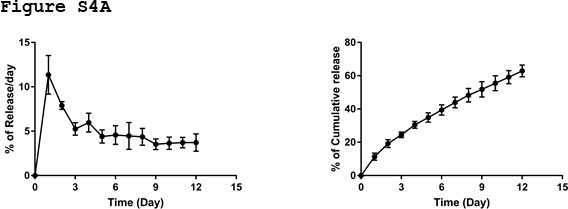


**B.** Release profile of IDL.


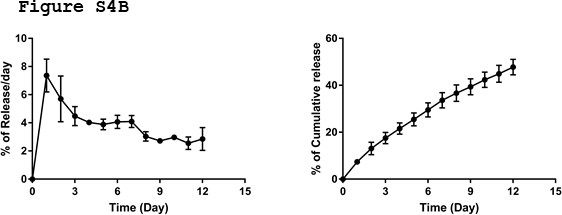


**Figure S5:** Cytotoxicity of blank NPs on HEK-293 cells


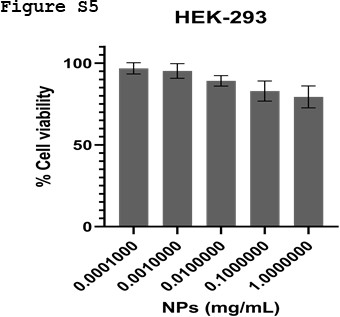


**Figure S6:** Replicate image of western blots


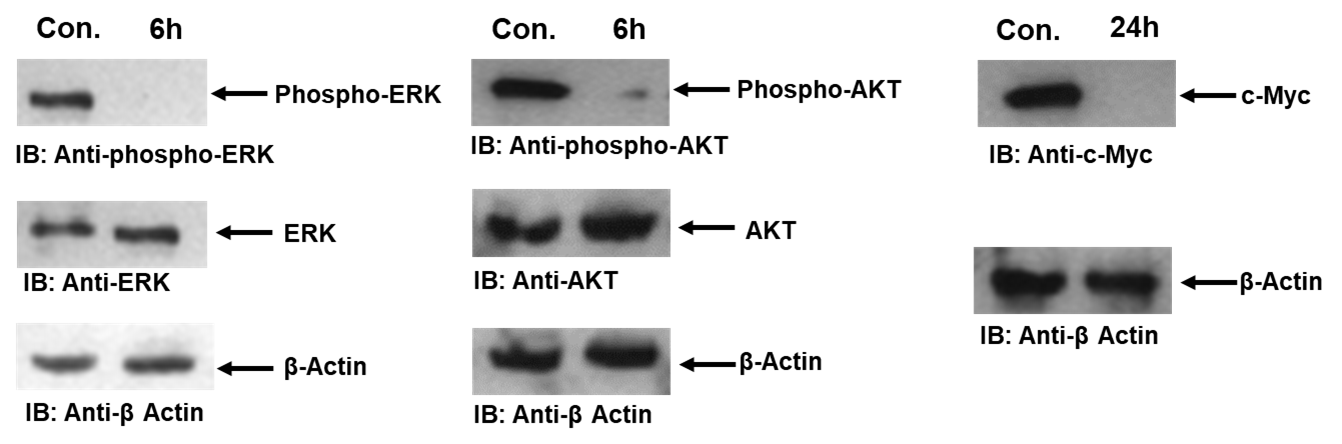


**Figure S7:** In-vivo distribution of free ICG and ICG NPs at 3h time point


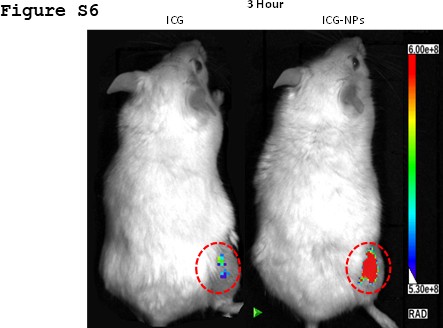


**References**

**1.** Thakur A, Tawa GJ, Henderson MJ, et al. Design, synthesis, and biological evaluation of quinazolin-4-one-based hydroxamic acids as dual PI3K/HDAC inhibitors. *Journal of medicinal chemistry.* 2020;63(8):4256-4292.

**2.** Fischer AH, Jacobson KA, Rose J, Zeller R. Preparation of slides and coverslips for microscopy. *Cold Spring Harbor Protocols.* 2008;2008(5):pdb. prot4988.
